# In Vivo Imaging of Corneal Endothelial Dystrophy in Boston Terriers: A Spontaneous, Canine Model for Fuchs' Endothelial Corneal Dystrophy

**DOI:** 10.1167/iovs.15-18885

**Published:** 2016-07-25

**Authors:** Sara M. Thomasy, Dennis E. Cortes, Alyssa L. Hoehn, Allison C. Calderon, Jennifer Y. Li, Christopher J. Murphy

**Affiliations:** 1 Department of Surgical and Radiological Sciences, School of Veterinary Medicine, University of California, Davis, California, United States; 2 Department of Ophthalmology & Vision Science, School of Medicine, University of California, Davis, California, United States

**Keywords:** canine model, Fuchs' endothelial corneal dystrophy, optical coherence tomography, corneal endothelial dystrophy, in vivo confocal microscopy

## Abstract

**Purpose:**

Boston Terriers (BTs) have a greater prevalence of corneal endothelial dystrophy (CED), in comparison to other canine breeds. Similar to Fuchs' endothelial corneal dystrophy (FECD), this condition is characterized by endothelial cell degeneration with secondary corneal edema. This study assessed corneal morphology using in vivo confocal microscopy (IVCM) and Fourier-domain optical coherence tomography (FD-OCT) in BTs with and without CED.

**Methods:**

The corneas of 16 BTs with CED and 15 unaffected, age-matched BTs underwent clinical evaluation and were imaged using IVCM and FD-OCT. A two-sample *t*-test or Mann-Whitney rank sum test were used to statistically compare parameters between groups. Data are presented as mean ± SD or median (range).

**Results:**

Mean age did not significantly differ between affected and unaffected dogs at 10.0 ± 2.0 and 10.6 ± 2.4 years, respectively (*P* = 0.437). Females (69%) were overrepresented among the CED-affected dogs. In CED patients, IVCM demonstrated endothelial polymegathism and pleomorphism. Corneal endothelial density was significantly less (*P* < 0.001) in dogs with CED (1026 ± 260 cells/mm^2^) versus age-matched controls (2297 ± 372 cells/mm^2^). Fourier-domain OCT demonstrated a significant increase (*P* < 0.01) in central corneal and endothelium-Descemet's complex thickness in dogs with CED versus age-matched controls at 1019 (485–1550) or 536 (464–650) μm and 32 (22–56) or 25 (15–34) μm, respectively.

**Conclusions:**

Corneal endothelial dystrophy in BTs is a bilateral, adult-onset condition that shares many similarities with FECD. Thus, CED could serve as a spontaneous disease model to study the pathogenesis of and develop novel treatments for FECD.

Corneal disease is a leading cause of blindness worldwide and corneal transplantation is often required to restore vision. A primary indication for corneal transplantation is to treat Fuchs' endothelial corneal dystrophy (FECD).^1^ This disorder affects up to 4% of the population over 40 years of age in the United States,^2^ and is characterized by progressive loss of corneal endothelial cells, which maintain stromal deturgescence, and thus corneal transparency. Human corneal endothelial cells have a limited capacity to regenerate and once a critically low number is reached in FECD patients, corneal edema occurs concurrently with vision loss.^3^ Genetic factors,^4^ damage from oxidative stress,^5^ RNA toxicity,^6^ and unfolded protein response^7^ are implicated in FECD but the fundamental mechanisms responsible for this disease remain unknown.

In dogs, corneal endothelial dystrophy (CED) is a bilateral, primary degenerative process of endothelial cells. With disease progression, CED and FECD can result in corneal edema, bullous keratopathy, ulcerative keratitis, and increased risk of corneal infection, which severely compromises vision and comfort in affected patients.^8,9^ Boston Terriers (BTs) with CED have abnormalities on clinical examination, light microscopy, and scanning electron microscopy (SEM) that resemble FECD.^10^ Furthermore, CED has been reported most commonly in BTs although other breeds may be predisposed,^10^ an observation that is suggestive of an underlying genetic component. A canine model for FECD affords several advantages, including spontaneous disease inheritance without concurrent congenital anomalies and a relatively diverse genetic population that is exposed to the same epigenetic factors as their human owners. Furthermore, dogs represent a large-eyed animal model facilitating the development of relevant medical and surgical procedures and evaluation of outcomes.

While the clinical, histologic, and ultrastructural characteristics of CED in dogs have been described,^10^ the use of advanced anterior segment imaging to thoroughly characterize the stages of disease has not been previously reported. Optical coherence tomography (OCT) and in vivo confocal microscopy (IVCM) are increasingly used for diagnosis and monitoring of FECD. Specifically, Fourier-domain OCT (FD-OCT) characterized and measured Descemet's membrane for pre- and post-surgical monitoring of patients with FECD.^11,12^ Specular microscopy or IVCM is commonly used by physician ophthalmologists to diagnose and monitor endothelial density and morphology in patients with FECD.^13^ Thus, the purposes of this study were to assess corneal morphology and determine changes in corneal thickness and endothelial density over time using FD-OCT and IVCM, respectively, in BTs with and without CED.

## Methods

### Animals

This study was approved by the Institutional Animal Care and Use Committee at University of California-Davis (#17324; Davis, CA, USA) and performed according to the ARVO Statement for the Use of Animals in Ophthalmic and Vision Research. All dogs were BTs. Prior to study entry, all dogs underwent a complete physical examination; dogs that were systemically well enough to receive sedation were included. All dogs received a detailed ophthalmic examination, including digital slit-lamp biomicroscopy (Imaging Module IM 900; Haag Streit, Koeniz, Switzerland), handheld slit-lamp biomicroscopy (SL-15; Kowa American Corporation, Torrance, CA, USA), binocular indirect ophthalmoscopy (Keeler Instruments Inc., Broomall, PA, USA) using a 28 diopter (D) indirect lens (Volk Optical, Inc., Mentor, OH, USA), and Schirmer tear-test 1 (STT-1; Intervet, Inc., Summit, NJ, USA). Inclusion and exclusion criteria are detailed in the Table. None of the dogs received topical eye medications for at least 24 hours prior to entry into the study. Dogs were sedated with acepromazine (0.01 mg/kg) and buprenorphine (0.01 mg/kg) intravenously then placed in sternal recumbency for imaging; OcuSOFT Eyewash (Altaire Pharmaceuticals, Inc., Aquebogue, NY, USA) was applied as necessary to prevent corneal desiccation.

**Table:**
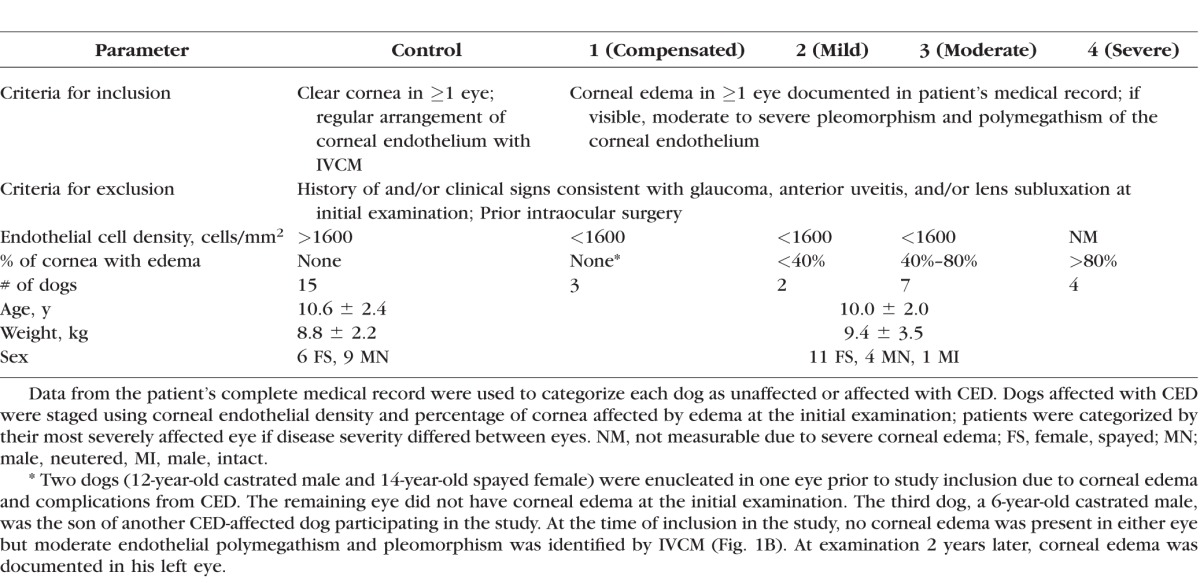
Criteria for Inclusion and Exclusion, Grading Scheme, and Signalment Data for Dogs Unaffected and Affected With CED

### Applanation Tonometry and Ultrasound Pachymetry (USP)

Intraocular pressure was measured by applanation tonometry (Tonopen XL; Medtronic Solan, Jacksonville, FL, USA) after applying 0.5% proparacaine (Alcon, Inc., Fort Worth, TX, USA) to the cornea. Ultrasound pachymetry (Accupach VI; Accutome Ultrasound, Inc., Malvern, PA, USA; and/or Pachette 3; DGH Technology, Inc., Exton, PA, USA) was performed in the central, superior, inferior, nasal, and temporal perilimbal cornea prior to FD-OCT measurements by placing the probe perpendicular to the cornea. Three and 25 consecutive corneal thickness measurements in each location were obtained and averaged automatically by the Accupach and Pachette, respectively, to obtain a mean value. All dogs had USP-Accupach performed; 13 CED-affected and 11 CED-unaffected dogs had USP-Pachette performed.

### Fourier-Domain Optical Coherence Tomography

At least 5 minutes elapsed between USP and FD-OCT measurements. Fourier-Domain imaging (RTVue 100, software version 6.1, 26000 A scan/sec, 5-μm axial resolution, 840-nm superluminescent diode; Optovue, Inc., Fremont, CA, USA) of the central cornea was performed using previously described methods.^14^ The RTVue measuring tool was used to measure central corneal thickness (CCT) and thickness of the epithelium, stroma, and Descemet's membrane (DM)-endothelium complex.

### In Vivo Confocal Microscopy

Next, IVCM (ConfoScan 4; Nidek Technologies, Gamagori, Japan) with a ×40/0.75 objective lens was used to image the central cornea. An eye gel of 0.3% hypromellose/carbomer 980 (GenTeal gel; Novartis, East Hanover, NJ, USA) was placed on the tip of the objective lens as an optical coupling medium and the lens was manually advanced until the gel contacted the central corneal surface. Automatic, full scans were performed without autoalignment and 350 images/scan were collected for each eye. Three repeat measurements were obtained of each structure measured. Superficial epithelial and endothelial cells were identified by their previously described appearance in the dog.^15^ Stromal images adjacent to epithelium and endothelium were used to identify keratocytes in the anterior and posterior stroma, respectively. Manual counts were performed using the ConfoScan 4 NAVIS imaging software. Three images per location were analyzed and results were averaged. For cell counts, cells touching the borderlines were counted only along one top or bottom border and one right or left border. Cells touching the opposite two borders were omitted from analysis.

### Grading for CED

All dogs were classified according to a grading scheme at the time of initial examination using endothelial cell density and percentage of cornea affected by edema (Table). If disease severity differed between eyes, dogs were classified according to their most severely affected eye.

### Longitudinal Study

The eyes of six affected and seven age-matched control BTs were assessed at the initial visit and approximately 1 year later using techniques described for the initial evaluation.

### Statistical Analysis

Eyes were analyzed separately and statistically compared for two measurements—endothelial density and percentage of cornea affected by edema for the longitudinal study. For all other measurements, values from each eye were averaged. A Student's *t*-test compared age, weight, epithelial thickness, keratocyte density, and endothelial density between groups; a Fisher's exact test compared sex distribution between groups. Least squares linear regression assessed the relationship between disease stage and corneal endothelial density or CCT as measured by FD-OCT. One-way analysis of variance (ANOVA) compared differences in total corneal thickness between groups by location. A Mann-Whitney rank sum test compared CCT, stromal, and DM-endothelium complex thickness between groups. For longitudinal data, a paired *t*-test compared endothelial density and percentage of cornea affected by edema in the less affected eye; a signed rank test compared percentage of cornea affected by edema in the worse affected eye. Data are mean ± SD or median (range).

## Results

### Study Population

**Figure 1:**
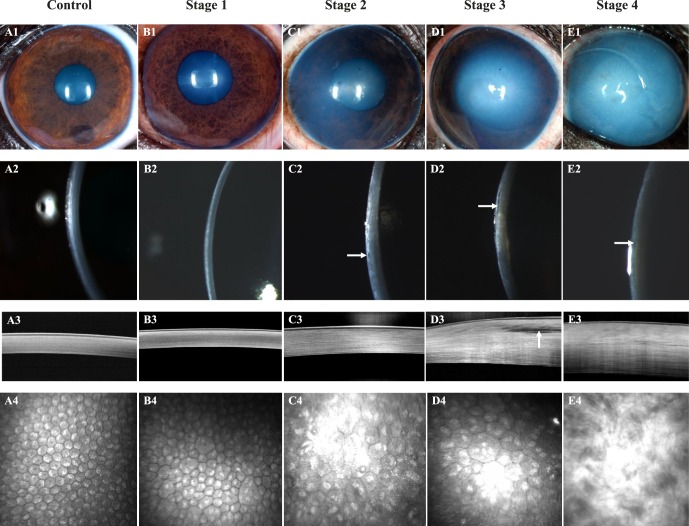
Corneal structure and cellular morphology as assessed by color photography, digital slit-lamp biomicroscopy, FD-OCT, and IVCM dramatically differed between control and CED-affected BTs with varying stages of severity. An 8-year-old castrated male BT with a clear cornea (**A1**, **A2**), normal corneal thickness, and lamellar arrangement of the collagen fibrils (**A3**), and regular, hexagonal arrangement of corneal endothelium (**A4**); this dog was categorized as a control. A 6-year-old castrated male BT with a clear cornea (**B1**, **B2**), normal corneal thickness, and lamellar arrangement of the collagen fibrils (**B3**) but moderate pleomorphism and polymegathism of the corneal endothelium (**B4**); this dog was the son of a CED-affected dog and was categorized as stage 1. An 11-year-old spayed female BT with mild edema in the temporal paraxial and perilimbal cornea (**C1**, **C2**) with microcystic bullae in the anterior stroma (**C2**, *white arrow*), increased corneal thickness (**C3**), and moderate pleomorphism and marked polymegathism of the corneal endothelium (**C4**); this dog was categorized as stage 2. An 11-year-old spayed female BT with moderate edema in the central, temporal paraxial and perilimbal cornea (**D1**, **D2**), increased corneal thickness with an axial anterior stromal bulla (**D2**, **D3**, *white arrows*) and marked pleomorphism and marked polymegathism of the corneal endothelium (**D4**); this dog was categorized as stage 3. A 14-year-old spayed female BT with marked, diffuse corneal edema (**E1**, **E2**), microcystic bullae in the anterior stroma (**E2**, *white arrow*), marked increase in corneal thickness (**E3**), and loss of the orderly arrangement of collagen fibrils within the anterior stroma (**E3**, **E4**), such that the corneal endothelium and keratocytes could not be visualized (**E4**); this dog was categorized as stage 4.

Twenty-nine eyes of 16 CED-affected BTs and 26 eyes of 15 unaffected BTs were included in the study. Three eyes (2 oculus dexter [OD] and 1 oculus sinster [OS]) of CED-affected dogs were enucleated and four eyes (2 OD and 2 OS) of 15 unaffected dogs were excluded due to extensive non-CED related corneal pathology. Age, weight, and sex distribution did not significantly differ between affected and unaffected dogs (*P* = 0.437, 0.582, and 0.156 respectively; Table). Disease severity varied between CED-affected patients (Table) from compensated with no change in corneal clarity (stage 1) to severe with diffuse, marked edema (stage 4, Fig. 1). Disease stage did not significantly correlate with corneal endothelial density (*P* = 0.217, *R*^2^ = 0.10) but did significantly correlate with CCT as measured by FD-OCT (*P* < 0.001, *R*^2^ = 0.66).

### USP

**Figure 2:**
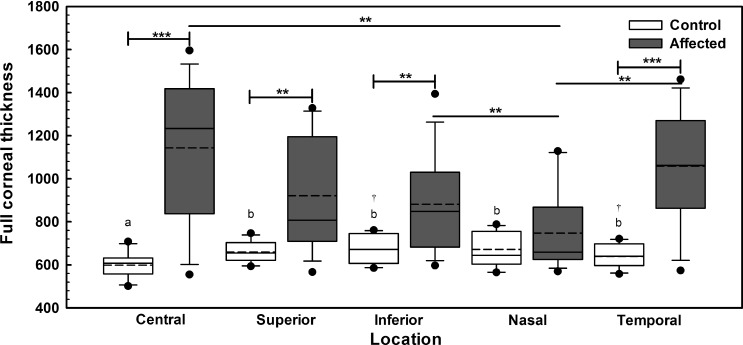
Full corneal thickness in 13 CED-affected dogs as measured by USP-Pachette dramatically differed in comparison to 11 unaffected controls. At the central, superior, inferior, and temporal locations, CED-affected dogs had significantly greater corneal thickness in comparison to control unaffected dogs. The nasal perilimbal cornea was significantly thinner than the central, inferior, and temporal locations in CED-affected dogs. The corneas of CED-unaffected dogs were significantly thinner centrally in comparison to the superior, inferior, nasal, and temporal perilimbal locations; temporal perilimbal cornea was also significantly thinner than the inferior cornea. *Box plots* depict median (*solid line*), mean (*dashed line*), 25th, and 75th percentiles, while whiskers show 10th and 90th percentiles; *black circles* indicate outliers. The *P* value was determined by a one-way ANOVA on ranks, **P < 0.01 and ***P < 0.001 between CED-unaffected and affected dogs or different locations of CED-affected dogs, and ^a,b,†^*P* < 0.003 between the different locations of CED-unaffected dogs.

All dogs had USP-Accupach performed but 13 (81%) CED-affected dogs had at least one region greater than the upper limit of the instrument (999 μm, data not shown). Using USP-Pachette, the corneas of CED-unaffected dogs were significantly thinner centrally in comparison to the superior, inferior, nasal, and temporal perilimbal locations with median values of 608 (502–708), 655 (594–747), 672 (585–761), 645 (565–788), and 640 (558–721) μm, respectively (*P* < 0.003, Fig. 2); inferior and temporal perilimbal corneal thickness significantly differed (*P* = 0.001). At the central, superior, inferior, and temporal locations, CED-affected dogs had significantly greater corneal thickness versus CED-unaffected dogs (*P* < 0.004, Fig. 2). Nasal perilimbal cornea was significantly thinner than the central, inferior, and temporal locations in CED-affected dogs with median values of 659 (570–1129), 1234 (555–1596), 849 (597–1394), and 1062 (574–1462) μm, respectively (*P* < 0.005, Fig. 2).

### Fourier-Domain Optical Coherence Tomography

**Figure 3:**
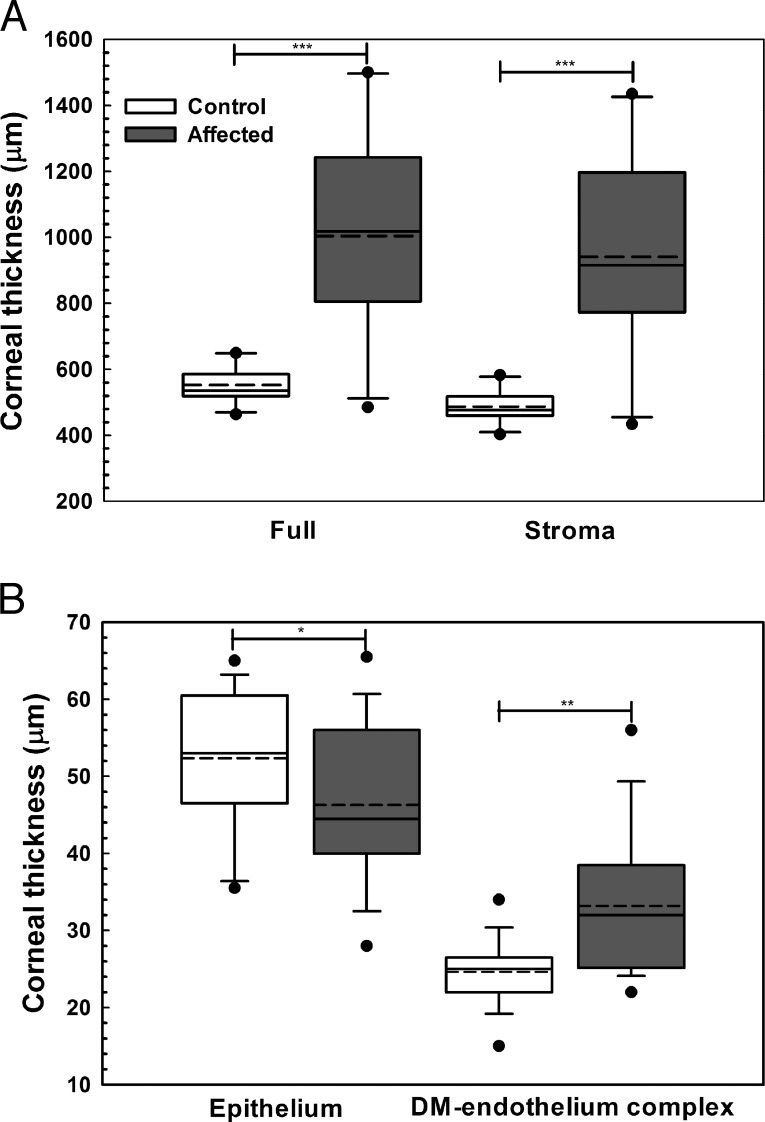
Central corneal full, stromal, epithelial, and DM-endothelium complex thickness significantly differed between CED affected and control (unaffected) dogs as measured by FD-OCT. Central corneal thickness was significantly greater in CED-affected versus unaffected dogs primarily due to a significant increase in stromal thickness in CED-affected versus unaffected dogs (**A**). Epithelial thickness in the central cornea was significantly less in dogs with CED versus control dogs (**B**). In the central cornea, DM-endothelium complex thickness was significantly greater in dogs with CED versus control dogs (**B**). *Box plots* depict median (*solid line*), mean (*dashed line*), 25th, and 75th percentiles, while whiskers show 10th and 90th percentiles; *black circles* indicate outliers. The *P* values were determined by a Mann-Whitney rank sum test (CCT, stroma, and DM-endothelium complex) or Student's *t*-test (epithelium), *P < 0.05, **P < 0.01, and ***P < 0.001.

In CED-affected dogs with corneal edema, corneal thickness increased with loss of the orderly arrangement of the collagen fibrils and an increase in reflectivity most apparent within the anterior stroma (Fig. 1). The CCT as measured by FD-OCT significantly differed between dogs with and without CED with a median (range) of 1019 (485–1500) and 536 (464–650) μm, respectively (*P* < 0.001, Fig. 3A). The difference in CCT observed was primarily due to a significant increase in stromal thickness in CED-affected versus unaffected dogs at 915 (434–1435) and 477 (403–583) μm, respectively (*P* < 0.001, Fig. 2A). Central corneal epithelial thickness was significantly less in CED-affected versus unaffected dogs at 44.5 (28–65.5) and 53 (35.5–65) μm, respectively (*P* = 0.039, Fig. 2B), while central DM-endothelium complex thickness was significantly greater in CED-affected versus unaffected dogs at 32 (22–56) and 25 (15–34) μm, respectively (*P* = 0.003, Fig. 3B). In CED-affected BTs versus age-matched controls, DM-endothelial complex was typically hyperreflective and hyporeflective, respectively (Fig. 1).

### In Vivo Confocal Microscopy

**Figure 4:**
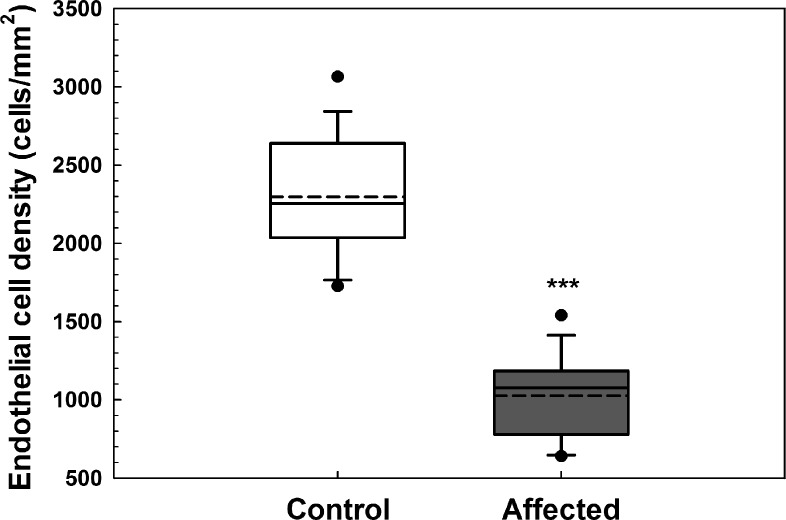
Endothelial cell density as measured by IVCM was dramatically lower in BTs with CED. Corneal endothelial density was significantly less in 21 eyes of 14 dogs with CED (1026 ± 260 cells/mm^2^) in comparison to 26 eyes of 15 unaffected controls (2297 ± 372 cells/mm^2^). Endothelial cell density could not be assessed in nine eyes of seven dogs with CED due to severe corneal edema (*n* = 6) or enucleation (*n* = 3); four eyes of control dogs were excluded from analysis due to non-CED related corneal pathology. *Box plots* depict median (*solid line*), mean (*dashed line*), 25th, and 75th percentiles, while whiskers show 10th and 90th percentiles; *black circles* indicate outliers. The *P* value was determined by a Student's *t*-test, ***P < 0.001.

**Figure 5:**
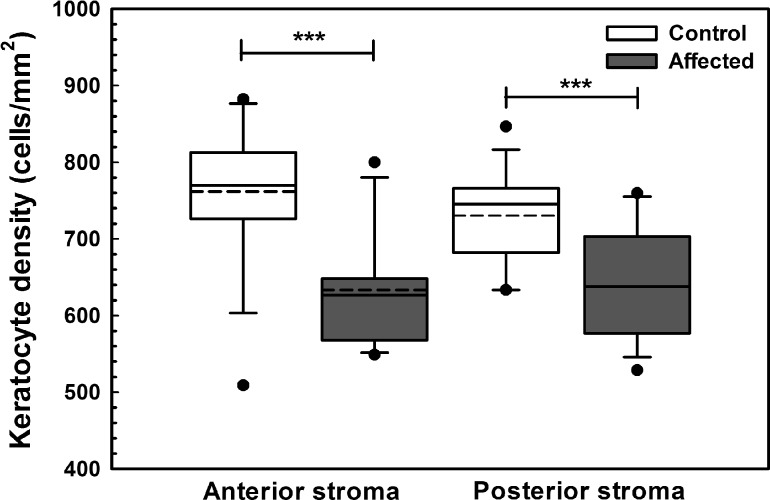
In the anterior and posterior stroma, keratocyte density as measured by IVCM was significantly less in BTs with CED in comparison to age- and breed-matched controls. Keratocyte density was significantly less (*P* < 0.001) in 13 dogs with CED (633 ± 73 cells/mm^2^) in comparison to 14 unaffected controls (762 ± 91 cells/mm^2^) in the anterior stroma. Keratocyte density was significantly less (*P* < 0.001) in 13 dogs with CED (638 ± 73 cells/mm^2^) in comparison to 14 unaffected controls (730 ± 61 cells/mm^2^) in the posterior stroma. Keratocyte density could not be assessed in three dogs with CED due to severe corneal edema and in one control dog due to poor quality images of the stroma. *Box plots* depict median (*solid line*), mean (*dashed line*), 25th, and 75th percentiles, while whiskers show 10th and 90th percentiles; *black circles* indicate outliers. The *P* values were determined by a Student's *t*-test, ***P < 0.001.

Obvious differences in corneal endothelial cell morphology were observed with a regular arrangement of hexagonal cells in unaffected dogs and polymegathism and pleomorphism in CED patients that increased with stage of disease severity (Fig. 1). Corneal endothelial density was significantly less (*P* < 0.001) in dogs with CED (1026 ± 260 cells/mm^2^) in comparison to unaffected controls (2297 ± 372 cells/mm^2^; Fig. 4). In the anterior and posterior stroma, keratocyte density was significantly less (*P* < 0.001) in dogs with CED (633 ± 73 and 638 ± 73 cells/mm^2^) versus unaffected controls (762 ± 91 and 730 ± 61 cells/mm^2^; Fig. 5), respectively. Endothelial cell density could not be assessed in six eyes of four dogs with CED due to severe corneal edema.

### Ancillary Diagnostic Tests

Aqueous tear production measured by STT-1 and IOP measured by applanation tonometry^16^ did not significantly differ between CED-affected and unaffected dogs at 23 ± 4 and 23 ± 3 mm/min and 10 (7–17) and 13 (6–18) mm Hg, respectively (*P =* 0.585 and *P* = 0.267, respectively).

### Longitudinal Study

Corneas of six CED-affected (4 spayed females, 1 castrated male, and 1 intact male) and seven CED-unaffected BTs (3 spayed females and 4 castrated males) were assessed at the initial visit and 10.7 ± 1.4 months later; no significant difference was detected between groups for sex distribution (*P* = 0.592). Mean age and weight at presentation did not significantly differ in affected and unaffected dogs at 10.7 ± 2.6 and 10.2 ± 1.5 years and 8.7 ± 1.8 and 9.0 ± 1.8 kg, respectively (*P* = 0.876 and 0.593, respectively). For CED-affected patients, disease severity of the six BTs were categorized as stage 1 (*n* = 1), stage 3 (*n* = 4) and stage 4 (*n* = 1).

**Figure 6:**
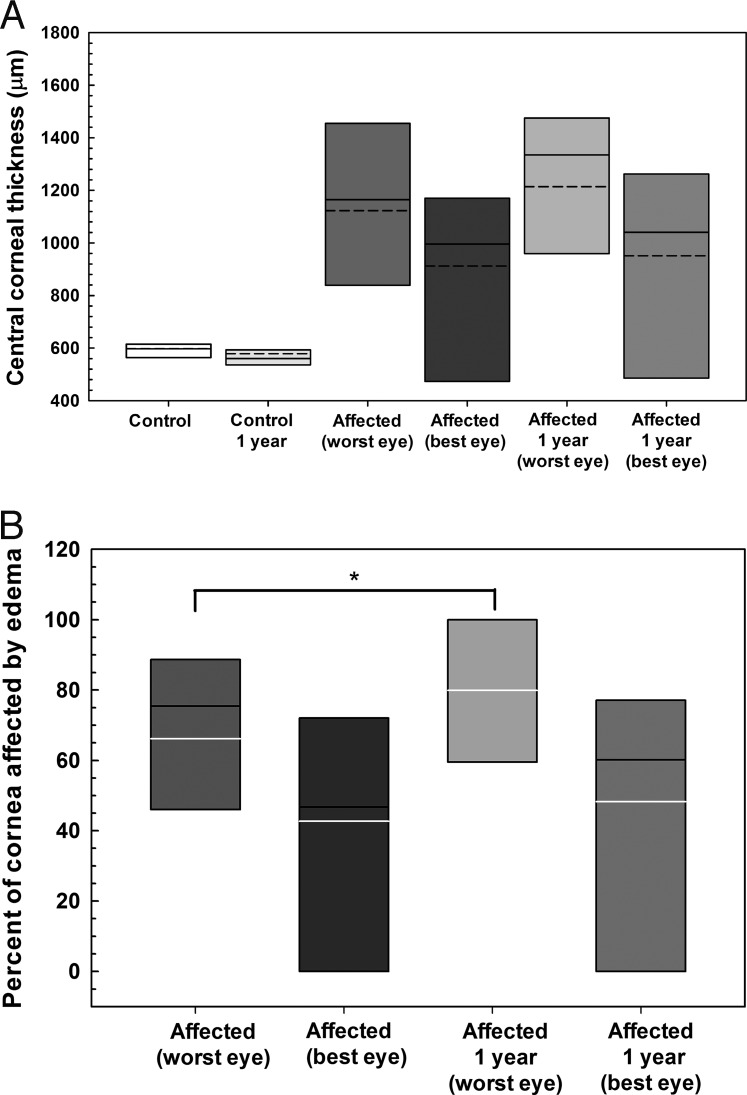
Central corneal full thickness did not dramatically change but percentage of cornea affected by edema significantly increased in the more severely affected eye over approximately 1 year in dogs without and with CED as measured by FD-OCT. (**A**) The CCT did not significantly differ (*P* > 0.05) over 10.7 ± 1.4 months in six CED-affected and seven unaffected dogs. (**B**) Mean ± SD percentage of cornea affected by edema significantly increased (*P* = 0.032) in the more severely affected eye of six dogs with CED from 66 ± 35% at baseline to 79 ± 40% at 10.7 ± 1.4 months. *Box plots* depict median (*solid line*), mean (*dashed line*), and 25th, and 75th percentiles. The *P* values were determined by a paired *t-*test for each group between the time points.

**Figure 7:**
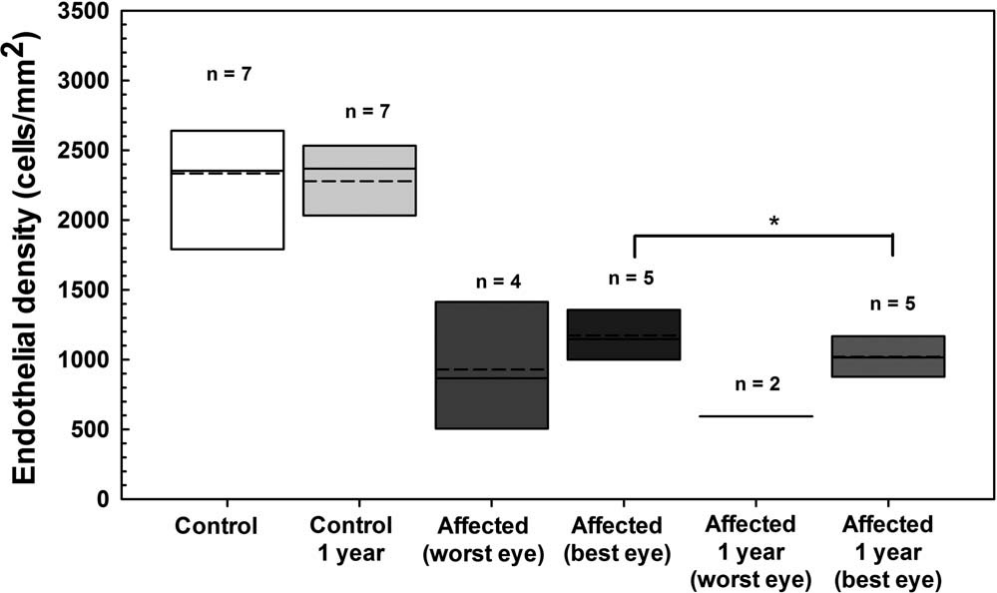
Endothelial density significantly decreased in the less affected eye of BTs with CED but did not change in control BTs (data averaged for both eyes) over approximately 1 year as measured by IVCM. Endothelial density did not significantly differ (*P* = 0.63) over 10.2 ± 1.2 months in seven unaffected dogs. Mean ± SD endothelial density significantly decreased (*P* = 0.043) in the less affected eye of five dogs with CED from 1172 ± 245 cells/mm^2^ at baseline to 1020 ± 161 cells/mm^2^ at 11.8 ± 1.2 months. Endothelial cell density could not be assessed in three eyes of two dogs and five eyes of four dogs with CED due to severe corneal edema at the onset of the study and at 11.8 ± 1.6 months, respectively. *Box plots* depict median (*solid line*), mean (*dashed line*), and 25th and 75th percentiles. The *P* values were determined by a paired *t-*test for each group between the time points. *P < 0.05.

Corneal thickness at the five locations measured did not significantly differ (*P* > 0.05) between baseline and 10.7 ± 1.4 months in six CED-affected and seven unaffected BTs using USP (Accupach or Pachette, data not shown). Similarly, CCT did not significantly differ (*P* > 0.05) over 10.7 ± 1.4 months in six CED-affected and seven unaffected dogs as measured by FD-OCT (Fig. 6). However, percentage of cornea affected by edema significantly increased (*P* = 0.032) in the more severely affected eye of six dogs with CED from 66 ± 35% at baseline to 79 ± 40% at 10.7 ± 1.4 months, while percentage of cornea affected by edema did not significantly differ (*P* = 0.25) in the less severely affected eye from 47% (0%–100%) at baseline to 60% (0%–100%) at 10.7 ± 1.4 months (Fig. 6). Corneal epithelial, stromal and endothelial-DM thickness did not significantly differ (*P* > 0.05) over 10.7 ± 1.4 months in six CED-affected and seven unaffected dogs as measured by FD-OCT (data not shown). Mean endothelial density significantly decreased in the less affected eye of five dogs with CED from 1172 ± 245 cells/mm^2^ at baseline to 1020 ± 161 cells/mm^2^ at 11.8 ± 1.2 months (*P* = 0.043, Fig. 7).

## Discussion

The results of the present study demonstrate numerous clinical similarities between canine CED and human FECD. First, CED is most prevalent in several breeds including BTs, Chihuahuas, and Dachshunds.^10^ This observation suggests that a heritable component for this disease may exist. A genetic basis for FECD is well established in human patients with mutations in *COL8A2*,^17,18^ *LOXHD1*,^19^ *SLC4A11*,^20^ *TCF4*,^21^ and *TCF8*^22^ reported to affect the collagen secretion or water pump functions of the endothelium.^23^ To the author's knowledge, there are no published reports evaluating the genetics of canine CED. In this study, we chose to evaluate the BT as this is the most common canine breed diagnosed with CED at the UC Davis Veterinary Medical Teaching Hospital (unpublished data) and is the 22nd most popular breed in the United States.^24^ In this study, mean age of CED-affected BTs at presentation was 10 years and consistent with previous reports of a middle-aged to older adult age of onset for CED in BTs.^9,10^ The age of presentation for CED in BTs is approximately equivalent to 55 years of age in a human,^25^ and thus very similar to the age of presentation for FECD of 50 to 60 years.^26^ In the present study, 69% of the CED-affected BTs were female; a female predisposition has previously been reported for canine CED.^10^ Similarly, a female preponderance is found in FECD-affected patients with females seen up to 3.5-fold more frequently than males and females exhibiting a more severe disease form.^27–30^

Using FD-OCT, we demonstrated that the DM-endothelial complex was significantly thicker and more hyperreflective in BTs affected with CED versus controls. The DM is also significantly thicker in FECD patients versus age-matched controls using ultra–high resolution OCT and light microscopy.^11,31^ Ultrastructural analysis of the endothelium and DM of BTs with CED using SEM demonstrated decreased numbers of endothelial cells as well as fibrous metaplasia, lamination of Descemet's membrane from fibrillar deposits, and posterior protuberances that could be consistent with guttae.^10^ A hallmark of FECD in humans is the formation of guttae or excrescences of extracellular matrix (ECM) on DM. Using IVCM, we did not observe obvious guttae in any CED-affected BTs evaluated in this study. However, it is possible that guttae form late in the disease course in CED-affected dogs and marked corneal edema prevents observation using IVCM or specular microscopy. Thus, further histologic and immunohistochemical studies of DM in BTs with CED are warranted to determine if guttae are present and to characterize the composition of the thickened DM. Even in the absence of guttae, the observed DM thickening in CED-affected BTs in combination with previous ultrastructural studies^10^ suggest that these dogs produce an abnormal ECM, a feature shared with FECD-affected patients.

In the present study, normal BTs exhibit a mean CCT of 536 μm, which is similar to the mean CCT of 527 μm reported in human patients aged 60 years and older.^32^ However, endothelial decompensation from canine CED results in a marked increase in CCT due to moderate to severe corneal edema with a 2- to 3-fold increase in CCT observed in most CED-affected individuals versus controls. By contrast, human patients with endothelial decompensation from FECD typically exhibit more subtle increases in CCT of approximately 10% to 50% versus normal individuals.^33^ This difference in CCT between canine CED and human FECD affected patients is likely multifactorial. First, key differences in corneal structure exist between humans and dogs. Dogs lack a Bowman's layer^34^ and exhibit markedly less collagen fiber intertwining throughout the stroma (unpublished data, Winkler M, Jester JV, Thomasy SM, Raghunathan VK, Murphy CJ) with a collagen fiber arrangement much more similar to rabbits than humans.^35–37^ Second, it is likely that human patients with mild corneal edema exhibit decreases in visual acuity that prompt rapid clinical evaluation and subsequent surgical intervention.^33^ By contrast, owners of dogs with canine CED are less likely to seek consultation with a veterinary ophthalmologist or choose a surgical intervention until late in the disease course due to blindness or ocular discomfort from recurrent corneal ulceration.^9,38^

Mean endothelial cell density was 1026 and 2297 cells/mm^2^ in CED-affected versus age-matched control BTs, respectively, a finding consistent with values from previous reports of a CED-affected BT^9^ and normal adult dogs,^39^ respectively. In addition, marked changes in endothelial morphology were observed in the CED-affected dogs with pleomorphism and polymegathism that increased with disease severity. While guttae were not identified in this study, changes in endothelial morphology appear similar between CED- and FECD-affected patients.^40^ Keratocyte density was 15% less for CED-affected versus control BTs in the anterior and posterior stroma. Multiple studies of FECD-affected patients have documented a decrease in stromal cell density, particularly in the anterior cornea.^41,42^ While the cause of keratocyte loss in CED- and FECD-affected patients is unknown, it may be a primary susceptibility to cell death^43^ or similar to stromal cell loss after epithelial injury.^44^ This decrease in keratocytes, the cells responsible for production and organization of stromal collagen, likely contribute to decreased transparency independent of edema observed in CED and FECD.^43^

Over an approximate 1-year period, mean percentage of the cornea affected with edema increased 20% in the more severely affected eye and mean endothelial cell density in the less severely affected eye decreased 13% in dogs with CED. Canine CED has the potential to be a valuable spontaneous in vivo model for FECD and specifically to test novel therapeutic compounds to slow disease progression and to evaluate novel surgical approaches to restore corneal transparency and improve visual function. It is suggested that prospective, masked, placebo-controlled clinical trials be performed to evaluate the efficacy of novel therapeutic approaches using dogs at a similar stage of disease severity. Increasing the number of dogs enrolled and measuring parameters at different time points for at least 1 year will also increase the probability of detecting a therapeutic benefit with a test article or surgical approach. Consistent with previous studies,^9,10^ we demonstrated that corneal decompensation in CED-affected BTs begins temporally and progresses to involve the entire cornea with nasal involvement occurring last. This initial temporal decompensation may be a result of increased oxidative stress to the corneal endothelium associated with ultraviolet light exposure as the nasal aspect of the canine cornea is partially protected by the third eyelid and shadowing by the nose. Oxidative stress appears to play a critical role in the onset and progression of FECD.^45^ In addition to measurements of corneal thickness and endothelial density, we suggest that digital photographs are acquired and pachymetry performed at multiple locations and different time points to measure percentage of the cornea affected by edema. We highlight that CED has an asymmetric onset and progression between eyes of individual patients, which introduces additional variability when conducting clinical trials. It is recommended that canine patients entered into clinical trial receive test article or placebo in both eyes rather than one eye receiving test article and the opposite eye receiving placebo to control for variability between eyes.

The advantages of a spontaneous, large animal model for FECD are numerous. First, naturally occurring diseases such as CED in BTs likely better reflect the complex environmental, genetic, and physiological variation present within the human population versus the highly uniform genetic background and homogenous environment characteristic of laboratory animals.^46^ Second, dogs are commonly used to investigate corneal diseases^47,48^ as well as for surgical interventions and outcomes especially with regard to keratoprosthetics^49^ due to their similar corneal anatomy in comparison to humans, relatively large eyes, and ease of handling in the laboratory. Endothelial injury models have been described in the rabbit, primate, and cat using transcorneal freezing,^50,51^ or surgical removal.^52^ However, these models lack the ECM abnormalities intrinsic to FECD. The most well characterized FECD models are two a2 collagen VIII (*Col8a2*) knock-in mouse models, which exhibit guttae and a decrease in endothelial cell density.^53,54^ Both murine models have been used to investigate the therapeutic efficacy of lithium^55^ and n-acetylcyteine.^56^ However, the use of murine models are limited for novel pharmacologic, surgical, and cell-based interventions as well as gene therapy where a larger eye is more optimal to evaluate therapeutic efficacy.^46,57^ Similarities in corneal thickness and structure suggest that pharmacokinetics would be more predictive for a canine versus murine model.^58,59^ Thus, veterinary clinical trials of CED-affected patients may provide a relevant, predictive translational step in advancing novel FECD treatments from the benchtop to human clinical trials.^42^

In conclusion, we have described the in vivo advanced ocular imaging characteristics of CED in BTs and demonstrated that CED is a bilateral, adult-onset condition, which shares many clinical similarities with FECD. Thus, CED could serve as a potent spontaneous disease model to study the pathogenesis of and novel therapies for FECD.
